# Aeroponic systems: A unique tool for estimating plant water relations and NO_3_ uptake in response to salinity stress

**DOI:** 10.1002/pld3.312

**Published:** 2021-03-29

**Authors:** Endale Geta Tafesse, Moses Kwame Aidoo, Naftali Lazarovitch, Shimon Rachmilevitch

**Affiliations:** ^1^ French Associates Institute for Agriculture and Biotechnology of Drylands Jacob Blaustein Institutes for Desert Research Ben‐Gurion University of the Negev Midreshet Ben‐Gurion Israel; ^2^ Department of Agro Enterprise Development Ho Technical University Ho Ghana

**Keywords:** abiotic stress, aeroponic, nutrient uptake, tomato, water relations

## Abstract

The study of transpiration, water, and nutrient uptake during abiotic stress in the root zone is hindered because of the hidden nature of the root zone. In this study, a modified aeroponic system was used to evaluate whole plant transpiration, nitrate and water uptake in the growth and development of tomato plants in response to salinity. Tomato seedlings were exposed to three levels of salinity (1.5, 4.5, and 9 dSm^−1^) and three levels of nitrate (1, 4, and 8 mM NO_3_) in a separate experiments conducted concurrently. Whole plant transpiration, water and nitrate uptake were estimated. Our study revealed that ~30 to 35 days after treatment (DAT), water uptake rate per plant increased from a common initial rate of about 0.05 to 1.1, 0.6, and 0.4 kg/day at 1.5, 4.5, and 9 dSm^−1^ respectively. The NO_3_ uptake rates in tomatoes grown in 1 and 4 mM NO_3_ were 5.5 and 22% respectively, of the uptake of tomatoes grown in 8 mM NO_3_. The estimation of nitrate uptake and lower sensitivity to salinity stress in the aeroponic showed the effectiveness and cost efficiency of the system in the cultivation of vegetables during abiotic stresses. The novelty of the system described is the continuous estimation of root and nutrient uptake by the whole plant at any given time.

**Abbreviations**
DAT: days after treatment
EC: electrical conductivity

## 1 INTRODUCTION

Aeroponics is a plant culture technique employed for the growth of plant where the roots are either continuously or periodically misted with a nutrient solution; it may be regarded as a variant of hydroponics where plant roots are constantly cultivated in a nutrient solution (Rubanenko & Hilitsky, 2011). Aeroponics has long been used as a research tool in root physiology (Barak et al., 1996) and is recommended as a technique for steady‐state control of nutrients, gas exchanges, root temperature, and moisture (Aidoo et al., 2017; Zobel et al., 1976).

The use of aeroponic systems as a research tool has demonstrated the absence or insignificance of an additional stress associated with stress of interest, for example hypoxia, under/over irrigation, and deposition of salt in the root zone which might be associated with application of salinity in the soil medium. The mechanical force that impaired the development and growth of roots is also minimized (Peterson & Krueger, 1988). Another advantage of aeroponic systems is their easy‐to‐use method of monitoring plant nutrition, pH, and electrical conductivity (EC; Tafesse, 2014). This type of system also supports continuous measuring of plant shoots and roots in both non‐destructive and destructive methods for analytical analysis in the laboratory (Aidoo et al., 2017). Aeroponic and hydroponic systems are both soilless plant cultures. In comparing soil and soilless systems, aeroponic systems allow the direct observation of plants without disturbances, so that necessary actions can be taken before any problem becomes irreversible.

However, aeroponics has not been more extensively used because of incomplete knowledge about the operational parameters, and the difficulty in maintaining the operating system (Weathers & Zobel, 1992). The absence of substrate buffering the root zone in the system makes plant vulnerable to total collapse within a relatively short period under electrical power outages and/or technical failure. In addition, the system requires constant servicing operations, which may be costly. For instance, the pumps and misters require maintenance and may be prone to potential component failure (Aidoo et al., 2017). The failure or clogging of misters may restrict the plant's access to water, causing it to lose turgidity and wilt, which may be irreversible.

Transpiration rate is affected by the water vapor concentration in the surrounding air and the leaf temperature, as well as the boundary layer and stomatal resistances (Kubota, 2016). Plants transpire most of the water they absorb from the soil in exchange for CO_2_ they obtain from the atmosphere, affecting the process of photosynthesis. Understanding transpiration when plants are exposed to salinity and inadequate plant nutrition will contribute to breeding of highly efficient plants in response to stress.

It was, therefore, hypothesized that a well‐structured aeroponic system design will enhance our understanding and facilitate the estimation of the transpiration rate which allows water and nutrient uptake of the plant in response to salinity stress at the root zone. These parameters are difficult to estimate under other growth conditions such as hydroponics and soil. Using tomato plants, we demonstrated that aeroponic systems can be utilized as a tool for continuous measurement of plant water uptake during salinity and different levels of plant nutrition in the root zone. Most importantly, we also showed estimation of total plant transpiration during the growth period.

## 2 MATERIALS AND METHODS

### 2.1 Description of aeroponic system

**FIGURE 1:**
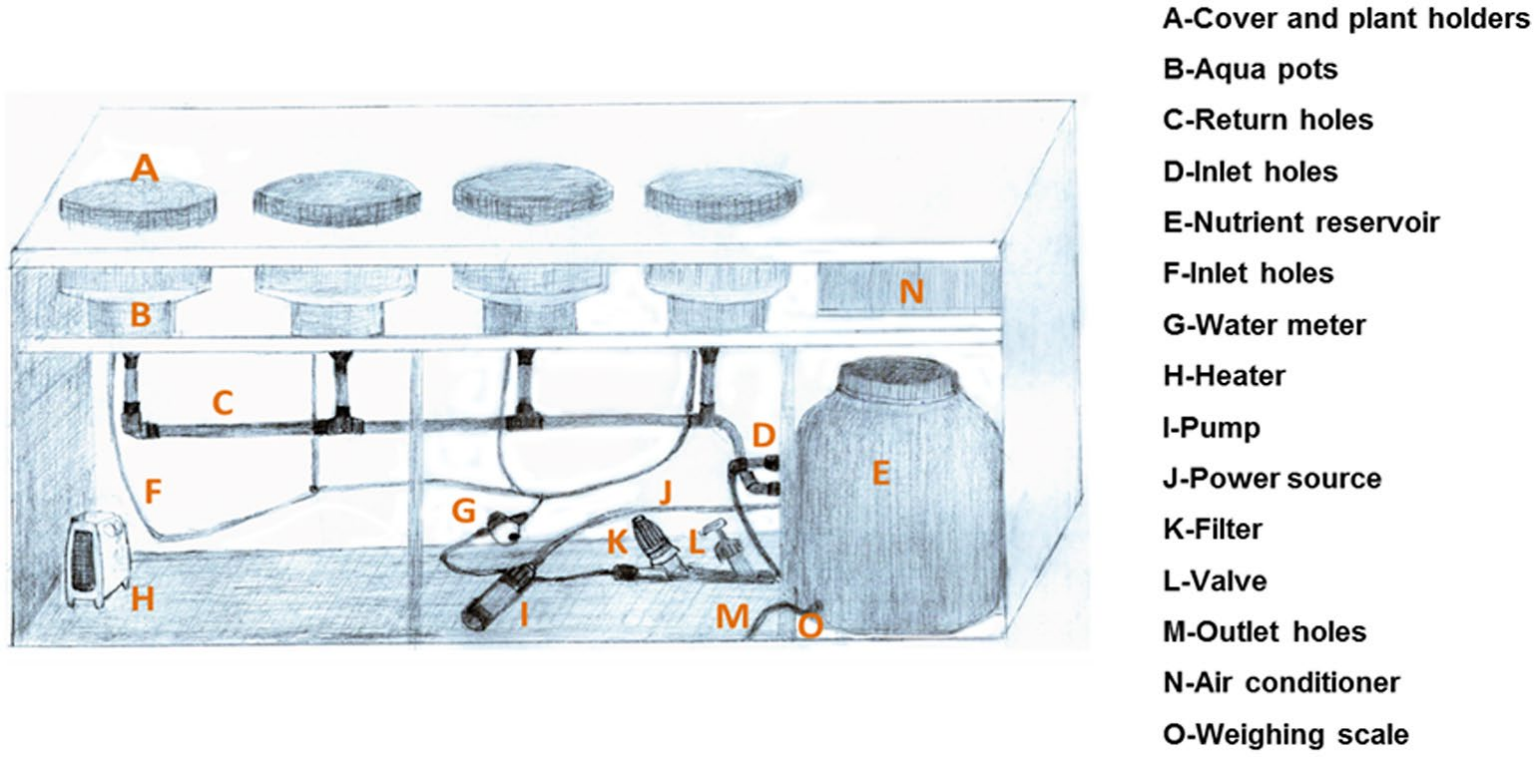
A sketch showing the aeroponic system, its accessories and various parts setup in a greenhouse located at Ben‐Gurion University, Sede Boqer Campus, Israel

Aeroponic systems are mostly, set up to control the injection of nutrient solution to the root zone for the growth of plants. These systems are mainly fixed with limited accessories for the delivering of nutrient solutions. In this study an aeroponic apparatus (Figure 1) consisting of circular aqua pots (Agro‐innovations LLC) made from polyvinylchloride (PVC) with a diameter of 50 cm and a depth of 14 cm was used. Within each aeroponic aqua pot, misters (Coolnet, Netafim) were installed to spray the desired fine mist directly onto the roots of the plants in the root zone. Four aqua pots were each fixed in the four large slots located on the top of the ion tanks (chambers) built with ion sheets on both sides insulated with Styrofoam to prevent influence from environmental factors during treatments. Built‐in air conditioners, heaters, thermocouples (Type E; not applicable to this study) and reservoir tanks to hold nutrient solution were installed in the chambers. The covers of the aqua pots were enforced with a thick black Styrofoam layer to create darker environmental conditions for the roots and to prevent algal growth and development inside the aqua pots. Nutrient reservoirs from which the nutrient solution can be directly drawn to the plant roots were placed on a digital scale interfaced to a computer via data loggers (CR1000, Campbell Scientific), and one additional reservoir, also located on a digital scale, served as the main source of the nutrient solution to the reservoirs located in the aeroponic chambers. A booster pump (Flojet) were used to draw the nutrient solution from the reservoir tank into the respective aeroponic aqua pots. The height difference between the aqua pot and the reservoir were used to return the solution to its respective reservoir after the roots were sprayed with the nutrient mist. In addition, EC meters (ES‐2, Decagon devices) were inserted into each of the solution reservoirs connected to data loggers monitoring the EC levels of the irrigation water. Built‐in notification alerts were activated in case there are mechanical faults in the operations of the system (Figure 1).

### 2.2 Plant materials, salinity, nitrate treatments, and experimental design

Two different experiments concurrently conducted in a six improved aeroponic systems three for each experiment (Figure 1) setup in the greenhouse at Ben‐Gurion University of the Negev, Sede Boqer Campus.

**FIGURE 2:**
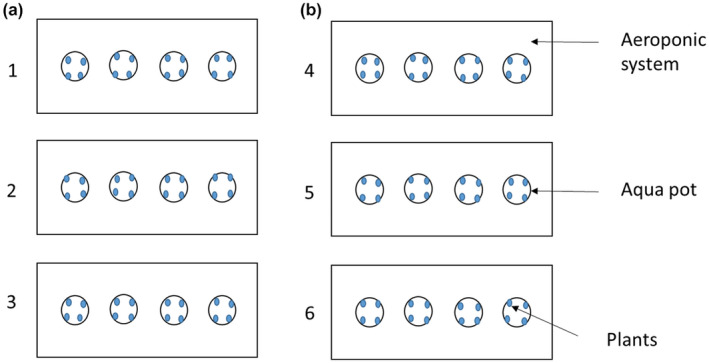
Experimental design setup in the greenhouse used for salinity (a; 1; 2; 3) and nitrate (b; 4; 5; 6) treatments. The design indicates the six aeroponic systems with four aqua pots each of the system and the number of plants per aqua pot (4) per aeroponic system (16)

A total of three different aeroponic systems were used for three salinity levels with electrical conductivity (EC) values of 1.5, 4.5, and 9 dS m^−1^ (thereafter, A1.5, A4.5, and A9) and a total of additional three levels of NO_3_ (1, 4, and 8 mM) in a separate experiments. Each treatment was assigned to a separate aeroponic system with four aqua‐pots fixed on top of it. An aqua‐pot takes 4 individual plants which sum up to 16 plants per treatment per aeroponic system. Six out of these 16 individual plants were measured as replicates in this study (Figure 2).

Seedlings of *Solanum lycopersicum* L. var. Mose were obtained from Zeraim Gedera Syngenta, Israel. After 30 days (end of July) of nursing the tomato seeds in a peat soil medium until they reached four fully produced leaves stage, the roots of the seedlings were gently washed and transferred to the aeroponic aqua pots, and after two weeks of acclimatization to the system, the plants were subjected to treatments.

Three reservoirs (for the three salinity levels) with a capacity of 0.2 m^3^ and two additional reservoirs with a capacity of 1 m^3^ were used in this study. The three reservoirs with a capacity of 0.2 m^3^, from which nutrient solution was directly drawn to the plants were placed on digital scales interfaced to a computer via data loggers (CR1000, Campbell scientific) so that continuous weight changes can be recorded and from which transpiration rate can be calculated. In the first experiment two big reservoirs with a capacity of 1 m^3^ were filled with the nutrient solution of 1.5 and 9 dSm^−1^ (A1.5 and A9, respectively) from which the three reservoirs were filled on a daily basis. The reservoir used for A1.5 and A9 were directly connected to their respective bigger reservoirs so that nutrient solution would draw into them based on a pre‐determined level of nutrient (weight of the tanks) whereas the reservoir for A4.5 (solution with EC 4.5 dSm^−1^) was filled by mixing solutions from the two big tanks by weight ratio. A4.5 and A9 desired salinity levels were obtained by applying NaCl in a half‐strength Hoagland nutrient solution (Hoagland & Arnon, 1950), which already had an EC of 1.5 dS m^−1^ hence A1.5 treatment. For A9, 36 mM of NaCl was applied in the half‐strength Hoagland nutrient solution, resulting in an EC of 9 dSm^−1^, whereas A4.5 was formulated by mixing the two solutions (A9 and A1.5) at mass ratio of 1:1.88 of A9 and A1.5, respectively, which resulted in an EC of 4.5 dSm^−1^. The NaCl was added proportionally to have an equivalent effect on the EC of the nutrient solution so that the competition between Na and K was minimized.

The second experiment involved in the evaluation of nitrate levels were also conducted in the three separated aeroponic systems (as explained above) with three levels of NO_3_ (1, 4, and 8 mM) added to the half‐strength Hoagland nutrient solution tested on tomatoes grown under salinity with EC of about 4.5 dSm^−1^ grown in an aeroponic system. Each level of NO_3_ was replicated six times as explained above during salinity experiment. In the preparation of NO_3_ stock solution, 202 g of KNO_3_ salt were mixed in a liter of water resulting 2 M KNO_3_ and 2 M Ca(NO_3_)2.4H_2_O were also prepared by mixing 472 g of Ca(NO_3_)2.4H_2_O salt in a liter of water. In the application of the treatments, 1 mM NO_3_ was made by applying 0.25 ml, each from stock solutions of KNO_3_ and Ca(NO_3_)2.4H_2_O, per liter of water applied to the plants. 4 mM NO_3_ was made by applying 1.0 ml, each from stock solutions of KNO_3_ and Ca(NO_3_)2.4H_2_O, per liter of water applied to the plants.

mM NO_3_ was made by applying 2.0 ml, each from stock solutions of KNO_3_ and Ca(NO_3_)2.4H_2_O, per liter of water also applied to the plants

The pH of the nutrient solutions was maintained between 6 and 6.5. In addition, EC meter sensors were inserted into each of the solution reservoirs and connected to data loggers so that the EC level of the water can be continuously monitored. The computer control spraying irrigate the plants for 1 min after every 6 min interval depending on the growth stage (size) of the plants and the ambient temperature. The ambient temperature recorded throughout the period of the experiment was 27 and 16°C (day and night temperatures respectively). The aeroponic chambers were subjected to the temperature of day light and other environmental factors in the greenhouse.

### 2.3 Estimation of transpiration and water uptake

Three reservoirs each with a capacity of 200 L, from which nutrient solutions were directly irrigated to the plants, were placed on digital balances interfaced to a computer via data loggers. Continuous mass change were recorded from which the transpiration rate was calculated based on the mass change of the nutrient solution on the scale due to plant uptake in relation to change in time. This method is technically similar to weighing lysimeters, but as there was no drainage in the closed aeroponic system and evaporation from the system was negligible, the mass decrease of the reservoirs was thus mainly due to plant water uptake. The mass of the tanks was recorded every 15 min.

### 2.4 Estimation of nitrate uptake

The plant nitrate uptake was estimated in the aeroponic system based on nutrient concentration depletion or uptake determined by calculating the differences between change in the volume of the nutrient on time points 1 and 2 multiplied by the nitrate concentration in the irrigated water solution on time point 1 and 2, respectively (Barak et al., 1996; Cabrera et al., 1995).

$$\text{Nutrient uptake rate}=\left(V1\times C1\right)‐\left(V2\times C2\right)$$

where *V*1 and *V*2 are the volumes of the nutrient solution at time points 1 and 2, and *C*1 and *C*2 are the nutrient concentrations (mmol/L) at time point 1 and 2. This gave an idea of the amount of nitrate taken up by the plant at any given time point. The nitrate concentration was then examined by ultraviolet absorption using a spectrophotometer (Epoch, BioTek) at 220 nm to obtain the nitrate reading and at 275 to correct the interference due to dissolved organic matter based on the method suggested by Greenberg et al., (1992). The nitrate concentration measurement was performed each day before and after the nutrient tanks were filled with fresh nutrient solution so that the amount of nitrate lost from the tanks due to plant uptake could be calculated.

### 2.5 Plant morphological characteristics

Growth parameters such as shoot fresh and dry weight, root fresh and dry weight including yield and fruit dry weight were measured at the end of the experiment thus after 90 days. Both below and above ground fresh weights were immediately determined whereas respective dry biomasses were determined after oven‐drying at 65°C for 72 hr.

### 2.6 Data analysis

Analysis of variance (ANOVA) and post‐hoc Tukey's HSD test were performed using statistica (version 11 Stat soft Inc.) to evaluate differences among the treatments for the measured plant parameters. The independent variables used as categorical predictors are salinity levels within each growth systems and the dependent variables were the measured plant parameters.

## 3 RESULTS AND DISCUSSION

This paper described the effectiveness of using an aeroponic systems regarding the measuring of whole plant transpiration rate, plant water, and nutrient uptake in response to salinity and different levels of nitrates. The provision of more accessories to enhance aeroponic system operations in this study, confirmed its effectiveness for experimental purposes (Figure 1). The use of aeroponic systems offers access to whole plant roots, preventing the loss of a significant portion of roots during washing from the soil. This system could also improve the roots taxa identification allowing species‐specific questions to be posed (Rewald et al., 2012).

**FIGURE 3:**
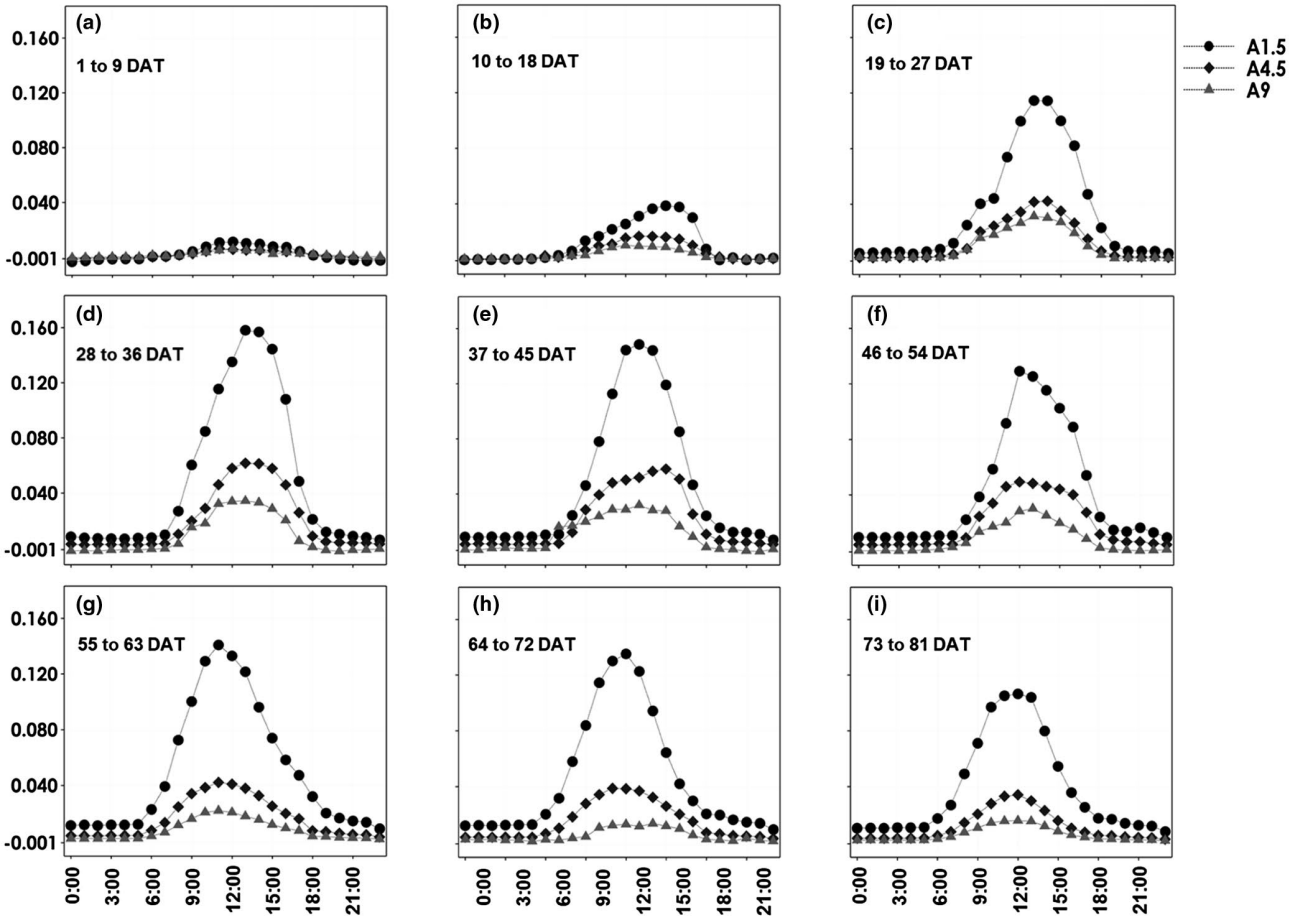
Diurnal pattern of water uptake rates in tomatoes under the three salinity levels (A1.5, A4.5, and A9) in the aeroponic system at different stages of plant growth based on days after treatment. The values mentioned at each point are the average of nine measurements from consecutive days taken at similar times with six replicates

To understand the rate of water loss, we measured the hourly transpiration rate of tomatoes, which varied throughout the day. These variations increased during the morning until the rate peaked around 12:00–15:00 hr of the day during the first two months (August and September) of the experiment (Figure 3a–d). In the last month of the experiment, the hourly transpiration peak was recorded around 9:00–12:00 hr of the days in October (Figure 3e–i). This might have been due to the end of summer pushing the transpiration demand to reach its peak earlier in the day as the days shortened. The maximum hourly water uptake rates per plant observed during 28–72 DAT during the afternoon were about 0.16, 0.08, and 0.03 kg for A1.5, A4.5, and A9, respectively (Figure 3d–h). However, at the beginning (1–27 DAT) and the last (73–81) hourly water uptake rates per plant recorded low values (Figure 3a–c,i). Such a large variation in the transpiration flux in a given day and during the entire plant growth period indicates the dynamic nature of the water uptake process and size of the plants, which is influenced by short and long‐term light and temperature and long term plant growth during the experimental period. The quick response of plants to such environmental changes have been highlighted by Munns (2002).

**FIGURE 4:**
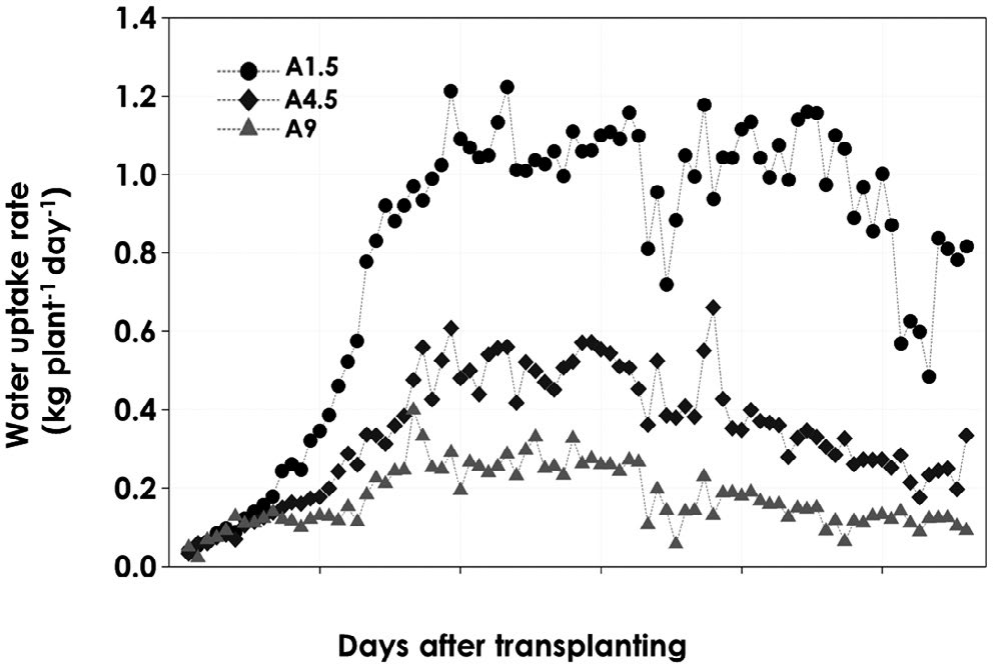
Water uptake rates per plant per day of tomatoes grown in an aeroponic system under the three salinity levels (A1.5, A4.5, and A9) over the entire growth period. Each point is the sum of 96 measurements recorded per day (recorded every 15 min) with six replicates. Variations in water uptake rate were first seen about 12 days after treatment (DAT)

In the average daily water uptake rate, 50 and 75% reductions were recorded in A4.5 and A9, respectively, compared to the control (A1.5) during peak transpiration periods (25–65 DAT). However, during the first ~15 DAT, no differences were observed between the three salinity levels. In the first ~30–35 DAT, the water uptake rate per plant increased from a common initial rate of about 0.05–1.1, 0.6, and 0.4 kg/day in A1.5, A4.5, and A9, respectively (Figure 4). Such an increase in the plant water uptake rate over time was mainly due to the active plant growth in terms of number and size of leaves, total leaf area, plant height, and total biomass accumulation as reported in Bhantana and Lazarovitch (2010).

**FIGURE 5:**
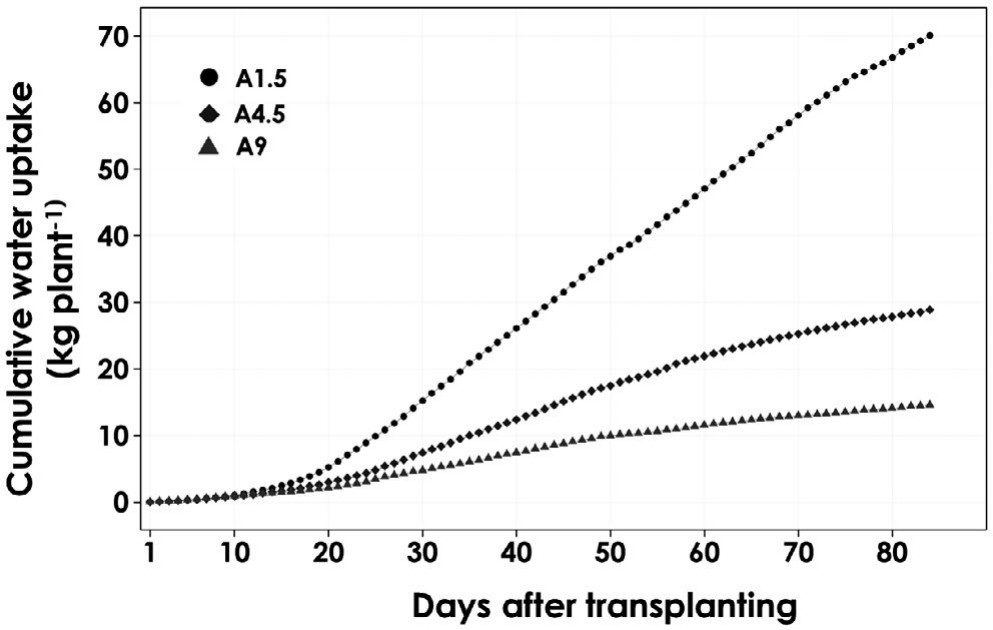
Cumulative water uptake rate per tomato plant grown in an aeroponic system under saline water (A1.5, A4.5, and A9) during the entire growth period. Values are averages of six replicates

The cumulative water uptake rates in each of the salinity level treatments were obtained by summing the respective daily uptakes (Figure 5). Similar to the hourly and daily rates, there was no significant difference between the salinity levels during the first 18 DAT, but at the end of the growth period, the cumulative transpiration rate of A1.5 was found to be more than two and five‐fold higher than A4.5 and A9, respectively. Generally, at 25 DAT, the increase in the cumulative transpiration rate was approximately linear with significant differences in the slope between the treatments. There was a significant reduction in the hourly, daily and cumulative transpiration rates due to salinity, and this finding is in agreement with a number of previous studies conducted on tomatoes and other crops’ responses to salinity (Maggio et al., 2007; Munns, 2002; Romero‐Aranda et al., 2001). Salinity mostly causes osmotic imbalances and reduced water uptake and transpiration, as well as reduced yields (Ben‐Gal et al., 2008). Increase influx and shoot accumulation of sodium has been linked to its toxicity and this has been reported to reduced biomass and yield significantly (Kronzucker et al., 2013).

**FIGURE 6:**
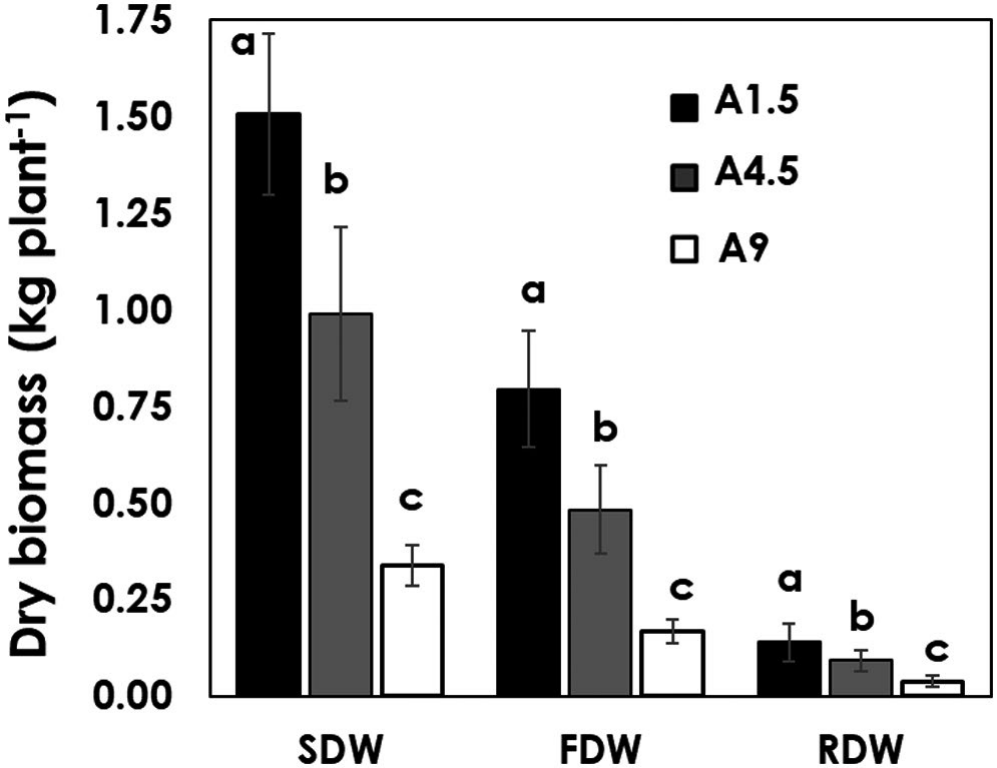
Shoot dry weight (SDW), fruit dry weight (FDW) and root dry weight (RDW), of tomatoes grown in aeroponic under three levels of salinity. Each value is an average of six measurements ± SE (six replication). Columns marked with similar letters compare across the treatments are not significantly different. (Post hoc Tukey test *p* < .05)

In measuring the plant biomass, significant differences (*p* < .001) were observed among the three levels of salinity stress. A1.5 treated plants had a higher total biomass than A4.5 and A9 (Figure 6). The reduction of total dry biomass was due to the increase of salinity treatments from EC 1.5 to 4.5 dSm^−1^. However, the biomass reduction (36%) in aeroponics was better compared to 55% reduction in the soil at similar salinity treatments reported by Tafesse (2014). This shows that perhaps plants grown in the soil is sensitive to salinity stress than aeroponics given the high deposition of Na in the root zone of the soil. This may be due to gradual build up and accumulation of salts in the root zone regardless of the leaching fractions in the soil. The reduced magnitude of biomass in the aeroponic system might have been due to enhanced uptake and utilization of water and nutrients even at a moderately higher level of salinity stress. The reduction of dry matter may be due to several adverse effects of salt stress such as specific ion toxicities, nutrient deficiencies, retardation of water uptake and nutritional imbalances in plants which affect enzymatic and physiological functions reducing growth, dry matter accumulation and yield of crops (Grattan & Grieve, 1999; Koyro, 2006; Mittler, 2006; Munns, 2002; Sagi et al., 1998).

**FIGURE 7:**
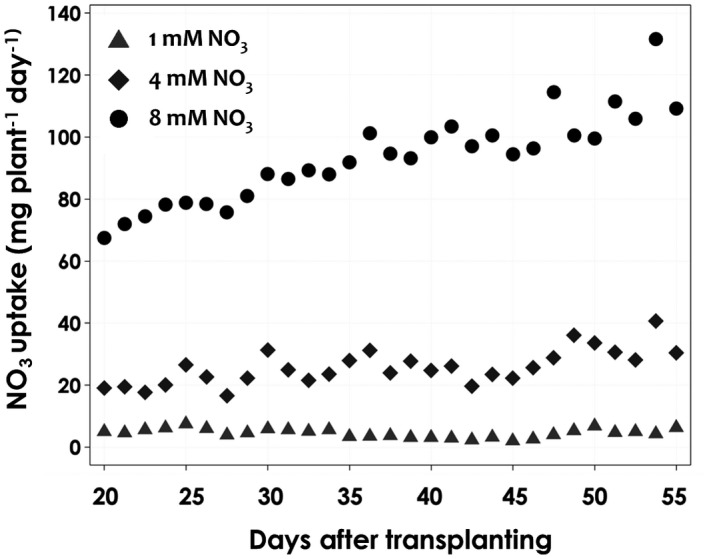
NO_3_ uptake of tomatoes grown under the three NO_3_ concentration (1 mM NO_3_, 4 mM NO_3_ and 8 mM NO_3_). The measurement was carried out for approximately 30 days from 20–55 DAT. Values are averages of six replicates

**FIGURE 8:**
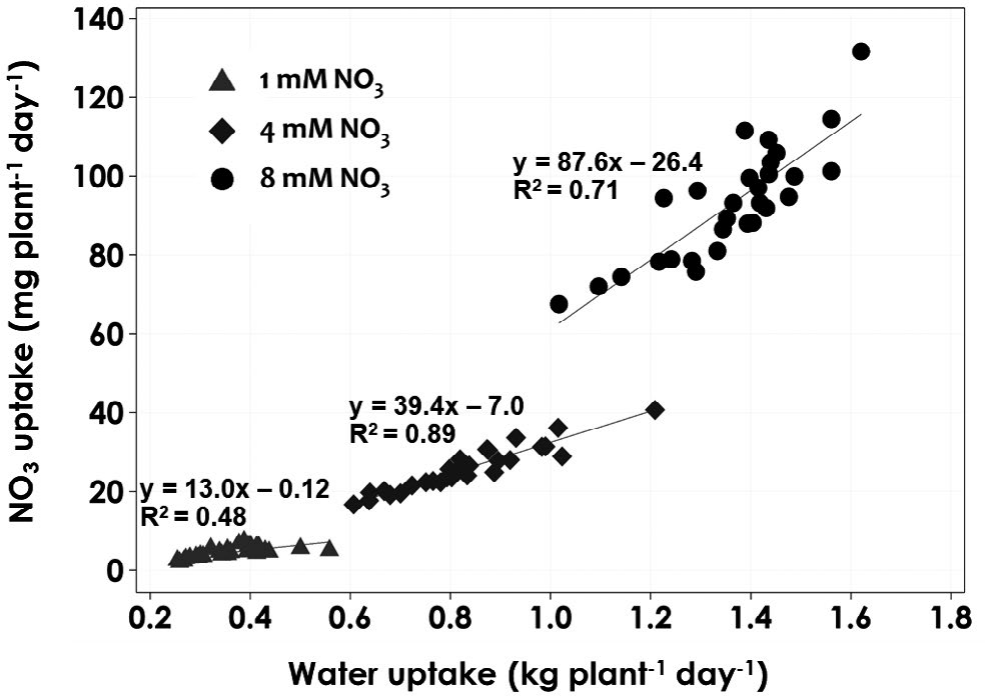
Relationship between NO_3_ uptake and water uptake of tomatoes grown under different NO_3_ concentration levels (1 mM NO_3_, 4 mM NO_3_, and 8 mM NO_3_) in an aeroponic system under saline conditions. Values are averages of six replicates

**FIGURE 9:**
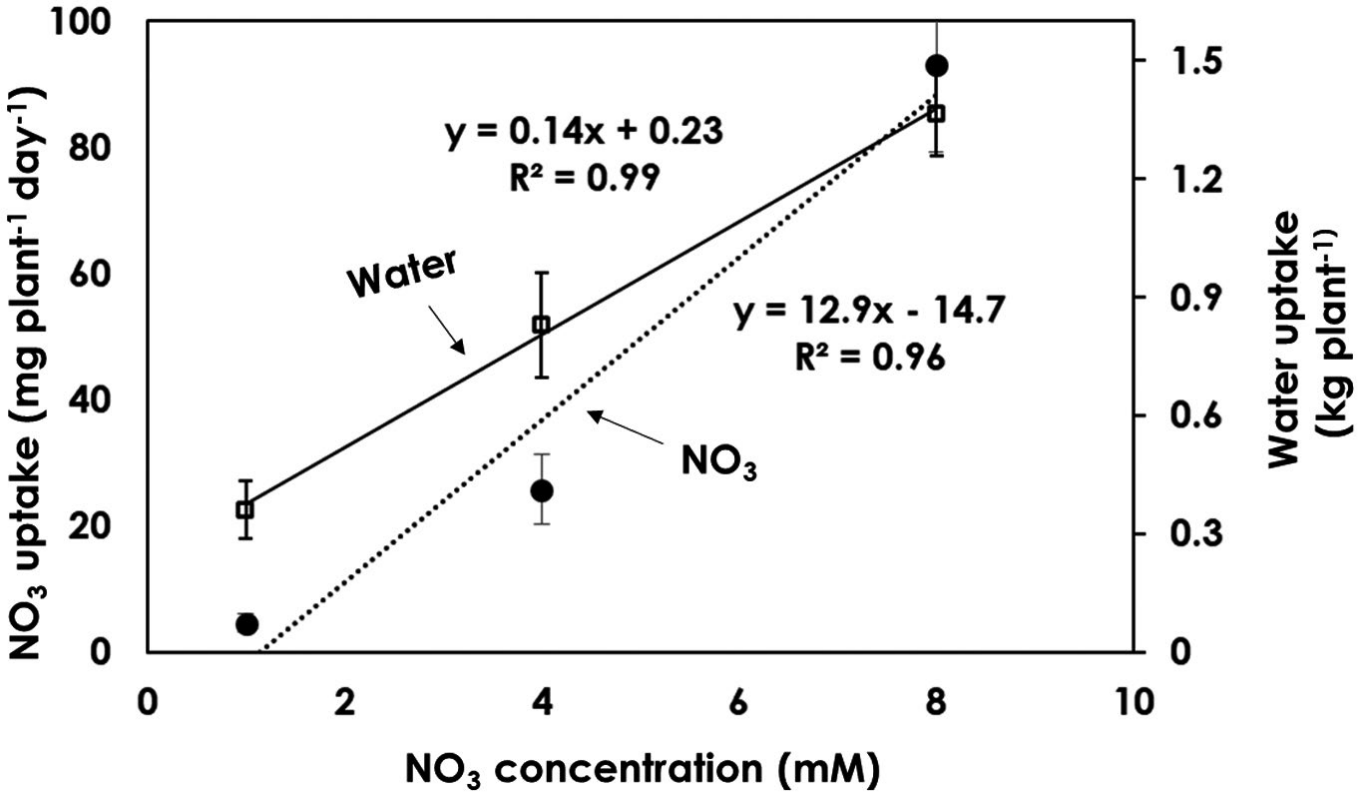
Relationship between NO_3_ uptake and water uptake of tomatoes as a function of concentration. The values presented here are the overall averages of NO_3_ and water uptake rates per plant per day during the entire growth period. Values are averages of six replicates

The NO_3_ uptake in the tomatoes treated with different NO_3_ concentrations showed significant differences between the treatments. The NO_3_ uptake rates of tomatoes grown in 1 and 4 mM NO_3_ concentrations were only 5.5 and 22% of the uptake of tomatoes grown in 8 mM NO_3_ (Figure 7). An increase in the NO_3_ concentration in the nutrient solution resulted in greater NO_3_ uptake (Flowers & Yeo, 1986), and this agrees with the finding that an increased N level in the solution significantly increases the N uptake in tomato plants’ shoot NO_3_ concentrations (Abdelgadir et al., 2005). The factor that limits a low uptake rate is the amount of nutrients available at the root surface, as reported by (Ingestad & Agren, 1988), in that the relative nutrient uptake rate is proportional to the relative plant growth rate. The nitrogen uptake rate is positively correlated with the plant water uptake rate, which is again affected by the plant growth rate (Figure 8). The regression line slopes for the three NO_3_ levels decreased with concentration. This indicates that the plant nitrate uptake rate is dependent on the nitrate concentration, and that a high concentration leads to a higher rate of nitrate uptake (Figure 9). In the study conducted by Abdelgadir et al., (2005), a close relationship was observed between cumulative transpiration and NO_3_ concentration in tomatoes.

**FIGURE 10:**
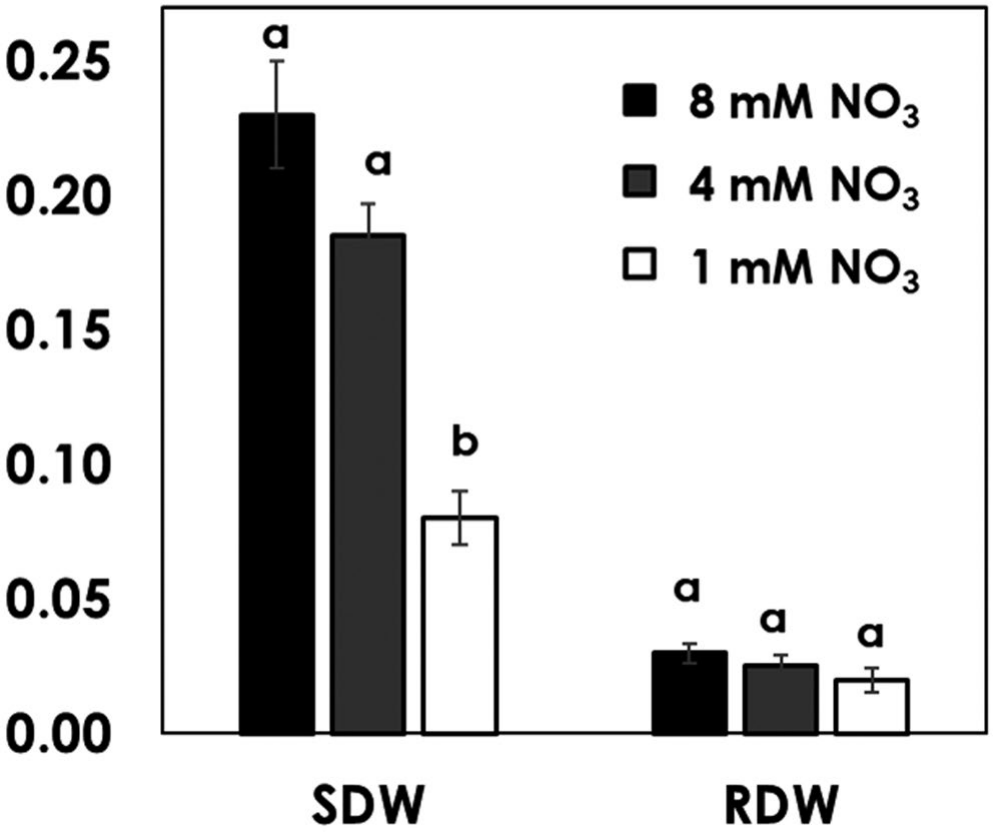
Biomass of tomatoes grown at the three levels of NO3 concentration expressed as shoot dry weight (SDW) and root dry weight (RDW). Columns marked with similar letters within each biomass parameter are not significantly different (Post hoc Tukey test *p* < .05). Error bars are standard errors (*n* = 6)

Below and above dry biomass were measured as shoot and root dry weight, and fruit yield. Different levels of NO3 concentration significantly (*p* < .05) affected plant biomass, except root dry weight (Figure 10). The higher the NO3 concentration, the higher the biomass obtained. The reduction of dry matter is as a result of reduced NO_3_ concentration from 8 to 4 mM (20%) and from 8 to 1 mM (64%). However, the difference between tomatoes supplied with 4 and 8 mM NO_3_ was not significant (Figure 10). Several studies showed progressive increase of dry matter accumulation with increase of NO3 levels (Abdelgadir et al., 2005). However, further increase of NO3 beyond the critical level, results in reduction in growth and biomass accumulation (Gastal & Lemaire, 2002) as well as shoot to root ratios (Bennett et al., 1989). The reason for the non‐significant difference in total biomass between tomatoes supplied with 4 and 8 mM NO_3_ is that the plants might have reached the NO_3_ critical content for maximum growth rate.

## 4 CONCLUSIONS

Aeroponic systems can be utilized for the fast, accurate and continuous measurement of plant responses to various environmental stresses especially in the root zone. Our findings have proven that in aeroponic systems, the root zone can be totally controlled during the experimental period. Irrigation can be adjusted at any time according to the demands of the plants, the treatments and the objectives of the study. Water and nutrient uptake by plants can be continuously and accurately measured at any stage of the plant growth and the experiment. Tomatoes grown in the aeroponic system were less sensitive to salinity. In aeroponics, nitrate supply to the plants can be minimized without significant reduction of the plant performances due to high nitrate uptake. The most interesting aspect of our findings is the continuous estimation of exact amount of water transpired by plant at any given time.

## CONFLICT OF INTEREST

The authors declare that they have no competing interests.

## AUTHORS’ CONTRIBUTIONS

EGT performed the experiment, analyzed data, and helped in writing the manuscript. MKA organized the data and helped in summarizing and writing the manuscript. NL designed the experiment. NL and SR supervised the discussion of the results. NL and SR were in charge of the research project. All authors read and approved the manuscript.

## Supporting information

Table S1
