# Supplementary figures and images for: Aeroponic systems: A unique tool for estimating plant water relations and NO3 uptake in response to salinity stress
Source: Plant Direct. 2021 Mar 29;5(4):e00312. doi: 10.1002/pld3.312 (PMC8006923; doi:10.1002/pld3.312)

Half strength Hoagland’s nutrient solution used


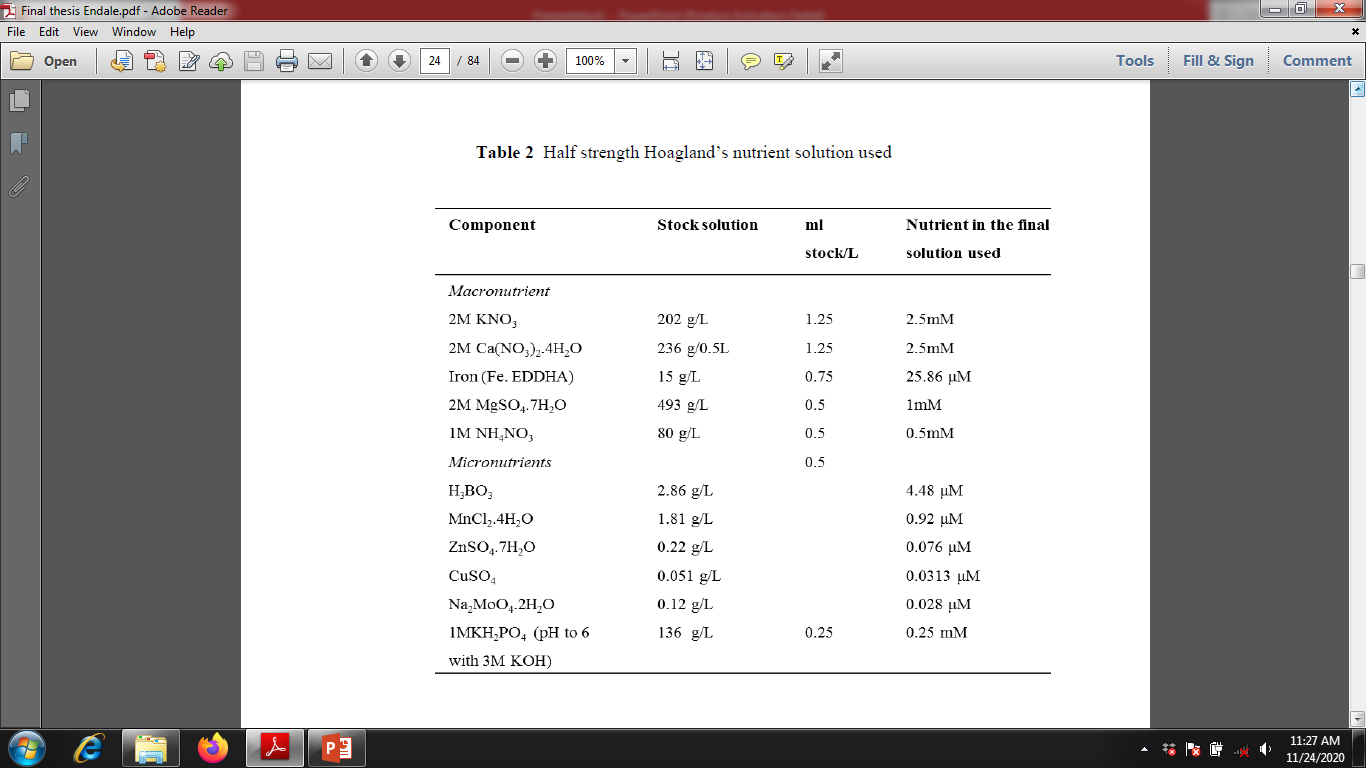

Supplement: Supplementary file 1 — Table S1 [file PLD3-5-e00312-s001.docx]
